# Smoking quit rates among patients receiving pharmacotherapy who received general practitioner counselling versus intensive counselling: a retrospective cohort study

**DOI:** 10.1186/s12875-022-01953-y

**Published:** 2022-12-27

**Authors:** Ilan Yehoshua, Limor Adler, Sharon Alon Hermoni, Miri Mizrahi Reuveni, Avital Bilitzky, Keren Oren, Galia Zacay

**Affiliations:** 1 Health Division, Maccabi Healthcare Services, Tel Aviv, Israel; 2 Department of Family Medicine, Ben-Gurion University of the Negev, Beer Sheva, Israel; 3 Department of Family Medicine, Sackler Faculty of Medicine, Tel Aviv University, Tel Aviv, Israel; 4 Bar Ilan University, Tel Aviv, Israel

**Keywords:** Smoking cessation, Varenicline, General practice, Counselling

## Abstract

**Background:**

Behavioral treatments can augment the success of pharmacotherapy in smoking cessation. The aim of this study was to compare smoking quit rates between patients receiving individual counseling with their general practitioner during office visits or intensive counselling with behavioral support, both augmented by varenicline.

**Methods:**

A nationwide retrospective cohort study conducted in a large Healthcare Maintenance Organization in Israel. We selected randomly patients who filled a prescription for varenicline and received either individual consulting by their general practitioner or intensive counselling with behavioural support, and asked them to answer a questionnaire. The outcome variables were smoking cessation 26–52 weeks following the beginning of treatment and satisfaction with the process.

**Results:**

870 patients were contacted and 604 agreed to participate (a response rate of 69%); 301 patients in the general practitioner group, 300 in the intensive counselling group and 3 were excluded due to missing date. The quit rate was 36.5% in the general practitioner group and 42.3% in the intensive counselling group (*P* = 0.147). In a logistic regression analysis, controlling for age, gender, socioeconomic status, ischemic heart disease, chronic obstructive pulmonary disease, pack years and duration of varenicline consumption, the adjusted OR for quitting in the general practitioner group was 0.79 (95% CI 0.56,1.13). The adjusted OR was higher in the group with the highest socioeconomic status at 2.06 (1.39,3.07) and a longer period of varenicline consumption at 1.30 (1.15,1.47). Age, gender and cigarette pack-years were not associated with quit rate. In the general practitioner group 68% were satisfied with the process, while 19% were not. In the intensive counselling group 64% were satisfied and 14% were not (*P* = 0.007).

**Conclusion:**

We did not detect a statistically significant difference in smoking quit rates, though there was a trend towards higher quit rates with intensive counselling.

## Background

As the leading cause of early death and preventable diseases worldwide, smoking is a global concern [1, 2]. The worldwide prevalence of daily smoking is 25%, [3] compared to a smoking rate of 20.5% in the adult population in Israel [4].

### Therapeutic modalities for smoking cessation

Therapeutic modalities for smoking cessation include behavioral and pharmacological therapy. Behavioral treatment options include brief guidance by the general practitioner (GP), intensive counselling workshops (usually moderated by nurses), personal telephone consultations, and web-based programs. A Cochrane review from 2021, that assessed the effect of behavioral interventions to support smoking cessation, concluded that all forms of counselling were effective, with more pronounced effect in the absence of pharmacotherapy [5]. Drug options include varenicline, an α4β2 nicotinic acetylcholine receptor partial agonist, sustained release bupropion, a norepinephrine and dopamine reuptake inhibitor, and nicotine replacement therapy (NRT) [6]. Behavioral treatment is recommended as support therapy for people using pharmacotherapy, [7, 8] as it increases quit rates by 10–20% [9].

### GP counselling vs. intensive workshops

There is a paucity of evidence regarding the effectiveness of brief counselling session provided by the patient’s personal GP, compared with intensive workshops. A randomized, controlled trial conducted in the Netherlands, compared these two modalities, both augmented with varenicline, and found similar quit rates with GP counselling, compared to nurse-moderated intensive workshops. The quit rates were 39% vs. 32.2% at 9–26 weeks following the intervention, and 28.8% vs. 25.5% at 9–52 weeks following the intervention, respectively [10]. A study from the US compared counselling by nurses with counselling by GPs and found a non-significant (NS) trend towards better quit rates with nurses (29.1% vs. 18.2%) [11]. A Cochrane review from 2017 compared individual counselling with no counselling (or brief counselling) and also more intensive counselling interventions with less intensive interventions [12]. Results showed that individual counselling is better than minimal contact control and that more intensive counseling is better than less intensive counselling. In both cases, all patients received NRT or bupropion.

### Smoking cessation in Israel

In 2010 smoking cessation workshops became available, free of charge, to all smokers in Israel, through all Israeli health maintenance organizations (HMOs) [13]. Varenicline and bupropion were offered at reduced cost to smokers who attended smoking-cessation workshops. Starting in 2015, NRT was also offered at a reduced cost as a second line treatment for smokers who attended the workshops. Starting in 2019, varenicline and bupropion were also available at reduced cost to smokers who were counselled by their GPs [14].

Over the past 10 years the number of patients attending intensive counseling workshops in Israel has increased by 230% from 11,844 persons in 2010 to 27,342 persons in 2017.

Maccabi Healthcare Services (MHS), the second largest HMO in Israel, offers different smoking cessation programs including eight 90-minute meetings in an intensive workshop, usually led by a nurse, personal telephone counselling delivered through six 30-minutes calls, and per-request GP smoking cessation counselling. GPs were invited to a short smoking cessation counselling training, in which they were advised to provide three smoking counselling sessions for each patient; however, it was not mandatory to provide all three. GPs were paid for smoking cessation counselling by the HMO once for each patient, regardless of the number of consultations that were actually done. We therefore assumed that each patient received between 1 to 3 sessions (the estimated duration of a session was 10–15 minutes). All these options can be augmented by pharmacotherapy, often with varenicline, which is the drug of choice because of its good efficacy and safety profiles [15, 16].

In light of the health policy change made in 2019, the aim of this study was to compare the effectiveness of smoking cessation by GP office visits (GP group) with intensive counselling (behavioral support; workshops or personal telephone support) (IC group), in both cases augmented by varenicline. The secondary aim was to compare patients’ satisfaction with the different programs.

## Methods

### Study design and setting

We designed a nation-wide retrospective cohort study using the MHS database. We identified patients who filled a prescription for varenicline from October 2019 to September 2020, and divided them to two groups according to the smoking cessation program they used: patients who participated in group workshops or received personal telephone sessions were included in the IC group, while patients who received smoking cessation counselling from their GP were included in the GP group.

Patients in both groups were selected randomly. We sent text messages to all patients 26 to 52 weeks after they first purchased varenicline, with a request to complete an online questionnaire to assess their smoking status. Text messages were sent in October 2020 and again in March 2021. Patients who did not respond were contacted by telephone between November 2020 and April 2021.

### Variables

The outcome variables were (a) quit rate, using an ordinal scale of 0–3 (0 – does not smoke, 1 – smokes rarely, 2 – smokes every day, but less than before, and 3 – smokes as much as before) and (b) satisfaction with the smoking cessation process using a 5-point Likert scale (1 = very dissatisfied, 5 = very satisfied). The quit rate was later reduced to a binary variable with patients who said they did not smoke at all categorized as non-smokers and the others categorized as smokers. Smoking status variable described the patient’s abstinence status from the pharmacological treatment until the time that the questionnaire was answered. Data for both variables were collected by patient self-report; no biochemical confirmation was obtained.

Behavioural support for smoking cessation, either intensive counselling or GP counselling, served as the exposure variable. Data were extracted from the electronic records and verified using patient self-report.

Confounder variables included sociodemographic data such as gender, age and socioeconomic status (SES); medical data including comorbid diseases such as diabetes mellitus type 2 (DMt2), hypertension, ischemic heart disease (IHD), chronic obstructive pulmonary disease (COPD), a history of malignancy; and smoking-related variables including cigarettes per day, duration of smoking in years and duration of treatment with varenicline in months. All sociodemographic and medical variables were obtained from the electronic medical records. All smoking related variables were obtained by patient self-report.

### Sample size

MHS proactively follows workshop participants with an 80% quit rate at the end of the workshop, and a 45% quit rate 1 year after the workshop. Accordingly, we assumed a quit rate of 45% in the IC group and considered a difference of 5% or less as non-inferior. 301 patients for each group were required for a significance level of 5% with a power of 80% in a 2-sided test. The sample size was calculated using the WinPepi software, v11.65.

### Statistical analysis

Descriptive statistics were used for all variables, with absolute numbers and percentages for categorical variables and mean and standard deviation for continuous variables. The chi-square test was used for univariate analyses of categorical variables. Student’s t-test was used for normally distributed continuous variables and the Mann-Whitney test for continuous variables which did not distribute normally. We performed a multivariate logistic regression analysis including variables with statistically significant associations with the outcome variables in univariate analyses, with the addition of smoking cessation group, age and gender regardless of the results of the univariate analysis. For null findings, we used the Bayes Factor to demonstrate that no association is a real finding and that the evidence supports the null hypothesis [17]. A Bayes Factor of less than 0.3 is considered substantial evidence to support the null hypothesis. We calculated a non-inferiority margin of 0.815, based on an assumption of 5% delta and a quit rate of 45% in the IC group. We used the Statistical Package for Social Sciences (SPSS) software version 27 for data analysis, and BayesFactor package in the R statistical programming environment to calculate the Bayes factor.

Ethical considerations.

All methods were carried out in accordance with relevant guidelines and regulations. Informed consent was given via the online questionnaire or orally during telephone calls. The local ethics committee (IRB) of MHS approved this study (ID 0062–20-MHS).

## Results

### Study population

From October 2019 to September 2020, 3817 patients fulfilled prescriptions for varenicline in MHS, 3066 (80.3%) received intensive behavioural support (by workshop or telephone), and 751 (19.7%) received GP counselling. We randomly sampled 435 patients from each group to participate in our survey. We sent the study questionnaire via text message and later conducted phone interviews with those who did not respond to the text messages. 604 patients (69%) agreed to participate in the study. Three patients were excluded due to missing data on behavioural counselling. Of the 601 patients who were included in the study, 300 were in the IC group and 301 in the GP group. The two groups were similar in gender and SES distribution, but patients in the IC group were older (47.4 vs. 44.7 years, *P* = 0.007) and smoked more (26.1 vs. 22.6 years, *P* < 0.001) (Table 1).

**Table 1:** Sociodemographic, clinical, and smoking-related characteristics of patients who underwent smoking cessation by intensive group counselling or by their GP

	IC group (***N*** = 300)		GP group (***N*** = 301)		***P*** value
	Count (%)	Mean (±SD)	Count (%)	Mean (±SD)	
**Sociodemographic data**					
Age		47.4 (±11.9)		44.7 (±12.4)	0.007
Gender					
Male	170 (56.7)		180 (59.8)		0.436
Female	130 (43.3)		121 (40.2)		
SES Group					
1–3 (low)	26 (8.7)		24 (8.0)		
4–7 (reference)	192 (64.0)		208 (69.1)		0.399
8–10 (high)	82 (27.3)		69 (22.9)		
**Presence of Chronic Diseases**					
Diabetes Mellitus type 2	31 (11.7)		27 (9.5)		0.388
Essential Hypertension	61 (23.1)		54 (18.9)		0.232
Ischemic Heart Disease	37 (14)		28 (9.8)		0.129
Chronic Obstructive Pulmonary Disease	12 (4.5)		11 (3.9)		0.689
History of malignancy	14 (5.3)		12 (4.2)		0.527
**Smoking related variables**					
Cigarettes per day		21.2 (±10.7)		19.6 (±9.0)	0.097
Duration of Smoking, years		26.1 (±12.0)		22.6 (±11.7)	< 0.001
Duration of consumption of Varenicline (months)		2.3 (±1.45)		2.2 (±1.45)	0.180

### Quit rates

The quit rate for the GP group was 36.5% and for the IC group 42.3% (*P* = 0.147). The crude odds ratio (OR) for success in the GP group was 0.78 (0.56, 1.09) (Table 2). A test of association yielded a Bayes factor of 0.117 in support of the conclusion that there is no relationship between study group and outcome.

**Table 2:** Univariate and multivariate analysis of characteristics of patients who stopped smoking

	Continued to smoke	Stopped smoking	Crude Odds Ratio (95% confidence interval)	Adjusted odds ratio (95% confidence interval)
	Count (%)	Count (%)		
**Smoking Cessation Group**				
GP	191 (52.5)	110 (46.4)	0.78 (0.56,1.09)	0.79 (0.56,1.13)
IC	173 (47.5)	127 (53.6)		
**Sociodemographic data**				
Age	46.6	45.14		
Mean (±SD)	(±12.26)	(±12.14)	0.99 (0.98,1.00)	0.99 (0.97,1.00)
Gender				
Male	217 (59.6)	133 (56.1)	ref	ref
Female	147 (40.4)	104 (43.9)	1.15(0.83,1.61)	1.21 (0.84,1.74)
SES Group				
1–3 (low)	36 (9.9)	14 (5.9)	0.70 (0.36,1.34)	0.81 (0.41,1.58)
4–7 (reference)	257 (70.6)	143 (60.3)	ref	ref
8–10 (high)	71 (19.5)	80 (33.8)	**2.02 (1.39,2.96)**	**2.06 (1.39,3.07)**
**Presence of Chronic Diseases**				
Diabetes Mellitus type 2				
Yes	40 (11.8)	18 (8.6)	0.70 (0.39,1.26)	
No	299 (88.2)	192 (91.4)	ref	
Essential Hypertension				
Yes	79 (23.3)	36 (17.1)	0.68 (0.44,1.06)	
No	260 (76.7)	174 (82.9)	ref	
Ischemic Heart Disease				
Yes	48 (14.2)	17 (8.1)	**0.53(0.30,0.96)**	0.59 (0.31,1.12)
No	291 (85.8)	193 (91.9)	ref	ref
Chronic Obstructive Pulmonary Disease				
Yes	10 (2.9)	13 (6.2)	2.17 (0.93,5.04)	2.46 (0.96,6.30)
No	329 (97.1)	197 (93.8)	ref	ref
History of malignancy				
Yes	17 (5.0)	9 (4.3)	0.84 (0.37,1.93)	
No	321 (95.0)	201 (95.7)	ref	
**Smoking related variables**				
Cigarettes per day				
Mean (±SD)	21.0 (±9.6)	19.4 (±10.3)	**0.98 (0.97,1.00)**	0.98 (0.97,1.00)
Duration of Smoking, years	25.1 (±11.8)	23.1 (±12.2)	**0.99 (0.97,1.00)**	
Duration of consumption of varenicline in months	2.04 (±1.2)	2.55 (±1.7)	**1.27 (1.14,1.43)**	**1.30 (1.15,1.47)**

In a logistic regression analysis, controlling for age, gender, SES, IHD, COPD, number of cigarettes per day and duration of varenicline consumption, no significant difference in quitting odds was found between the IC group and the GP group. The adjusted OR (aOR) for GP group quitting was 0.79 (0.56, 1.13) with a Bayes Factor of 0.1 (Table 2). Non-inferiority could not be proven and the result is inconclusive.

For the entire study population, significantly higher aORs for quitting were found in the highest SES group with an aOR of 2.06, (1.39,3.07) and with a longer duration of varenicline consumption with an aOR of 1.30 (CI 1.15,1.47). Patients with COPD had an insignificantly higher aOR of 2.46 (0.96,6.30). Age, gender, and number of cigarettes per day were not associated with quit rate.

### Satisfaction

In the entire study population, satisfaction was significantly higher for patients who stopped smoking (82%) compared with those who continued smoking (35%) (*P* < 0.001). In the GP group, 68% of the participants were satisfied (57% very satisfied and 11% satisfied), while only 19% were not satisfied or not satisfied at all. In the IC group 64% were satisfied (51% very satisfied and 13% satisfied) and 14% were not satisfied or not satisfied at all. The proportion of participants who were neither satisfied nor dissatisfied was higher in the IC group (23% vs 14%) (*P* = 0.007).

## Discussion

### Summary

We conducted a retrospective nation-wide cohort study to assess the difference in quit rates using two counselling styles, brief GP counselling during an office visit or intensive counselling, in both cases augmented by varenicline. We report a non-significant lower quit rate in the GP group compared with the IC group (36.5% vs. 42.3% respectively), with an aOR of 0.79 (0.56,1.13). Non-inferiority could not be proven, and the result was inconclusive. In the entire study group, we found a higher satisfaction rate for quitters compared with patients who continued smoking (82% vs. 35%) and for the GP group compared with the IC (68% vs. 64%).

### Strengths and limitations

The strengths of this study are its relatively large sample size, the high response rate (69%) and the heterogeneity of the participants and the large number of health providers personnel (GPs and nurses). Another strength is the observational design that provided real-world results, as the smoking cessation counsellors and the patients were not aware of being observed, they behaved as they usually do. This design eliminated a possible Hawthorne effect.

This study has several limitations. Being an observational study, compared with a randomised controlled trial study, exposes the study to selection bias. Patients who chose to attend an intensive counselling group might have had different characteristics from those who chose GP counselling. In order to overcome this bias, we compared the groups and checked for differences that might have impacted the outcomes, including comorbidities (Table 1). We also compared basic differences between patients who chose to attend the study and those who refused to take part (Table 3). In addition, we did not enquire regarding baseline psychological factors that might have influenced the outcomes. Another limitation is lack of uniformity of GP counselling, which could vary between merely providing a prescription to repeated counselling sessions. Although the study had a relatively large sample size, it was inadequate to prove a significant association (or lack of it) in quitting rates between the two groups. Last, as a retrospective study, the information which was provided in the questionnaire, including self-reported quitting status might have been biased.

**Table 3:** Age and sex of patients who participated in the study compared to patients who refused to participate

	Agreed to participate (***N*** = 601^**a**^)		Refused to participate (***N*** = 267)	
	Count (%)	Mean (±SD)	Count (%)	Mean (±SD)
Quitting method				
IC group	300 (49.9)		160 (59.9)	
IC group	301 (50.1)		107 (40.1)	
Gender				
Male	350 (58.2)		168 (62.9)	
Female	251 (41.8)		99 (37.1)	
Age		46.0 (±12.2)		47.4 (±12.3)

^a^ Six hundred and four agreed to participate; three were excluded due to missing data

### Comparison with existing literature

#### GP group vs. IC group

A similar study, from the Netherlands compared nurses-led intensive counselling to GP brief counselling. The nurses-led group included 3 face-to-face and 7 telephone sessions, mostly done by the first 13 weeks of the quitting attempt. In the GP group, patients had at least one visit for smoking cessation with their GP and received a prescription for varenicline. This study found a non-significant trend in favor of the GP group compared with a nurses-led group, with a quit rate of 39% vs. 32.2% respectively [10]. There are two differences between the Dutch study and the present one that might explain the opposite trends that were found: (a) the Dutch study was a randomized controlled trial, while the current study was a retrospective cohort, and (b) the Dutch study used chemical validation to check smoking abstinence while the current study relied on self-report. The lack of a significant difference in both the Dutch study and the present one suggests that these types of behavioral therapy support have a similar effect when augmented with varenicline.

A Cochrane review of eight RCTs (2048 patients), that compared more intensive to less intensive counselling, found a relative effect of 1.26 [95% CI 1.04–1.52] in favor of the more intensive counselling, however the patients in seven of these studies were treated with NRT, and with a combination of NRT and bupropion in one study, none were treated with varenicline [12]. The different result of the present study may result from the different pharmacological treatment.

MHS offers its patients intensive counselling groups free of charge, but they had to actively register for the workshop. On the other hand, GP counselling starts during a regular office visit with the patient’s personal GP, often initiated by the physician during an appointment set for other purposes. This may result in a selection bias where patients in the intensive counselling group are more likely to be at a more advanced Prochaska’s stage [18]. According to Prochaska’s model there are five stages in the change process: pre-contemplation, contemplation, and preparation for people who currently smoke; and action and maintenance for those who have quit. Interventions which require action, are more effective in the advanced stages [19–21]. On the other hand, in MHS the patients had long relationship with their personal GP, who usually knew them well, and had the opportunity to offer assisted quitting in good timing, and was also available for further counselling and support.

Another possible explanation for the differences in quit rates between the two groups is differences in patients’ characteristics in each group; the patients in the intensive counselling group were older and smoked more. In a recent cohort study, smoking cessation success was significantly associated with older age and fewer years of smoking [22]. No association was found with the number of cigarettes that were smoked per week. In our multivariate analysis, age and pack-years, which may be equivalent to years of smoking, were not associated with successful quitting.

Our study demonstrated a relatively higher quit rate for both groups, compared with previous publications on the effectiveness of varenicline [15, 16]. This could be related to self-reported quit status, without chemical validation of abstinence; however, it has been shown that self-reported smoking status was a reliable measure [23–28].

The multivariate analysis, demonstrated that patients with higher SES had a higher odds ratio for quitting, which is consistent with the results of previous studies [29–31].

#### Satisfaction with the process

We found greater satisfaction in the GP group, in contrast to the findings of the Dutch study, where a higher degree of satisfaction was seen in the nurses-led group. This difference may result from the different study designs, i.e., a retrospective cohort vs. a randomized control trial. Patients in the current study chose whether to actively attend a workshop, or to rely solely on the counselling of their GP, a possible source of selection bias that could have affected satisfaction from the process as well. The lower proportion of participants, who were neither satisfied nor dissatisfied in the GP group (14% vs 23%), might indicate a higher level of emotional engagement with their personal GP compared with a smoking cessation counselor, with whom they had no prior familiarity.

## Conclusion

This study did not find that office-visit smoking cessation counselling was significantly different from intensive behavioral therapy when augmented by varenicline. However, the results of non-inferiority were inconclusive. This finding should encourage GPs to deepen their knowledge regarding smoking cessation counselling and to offer this counselling during regular visits. Randomized controlled trials are required in order to further validate the results of this study in a controlled environment.

## Data Availability

The data that support the findings of this study are available from Limor Adler (the corresponding author) but restrictions apply to the availability of these data, which were used under license for the current study, and so are not publicly available. Data are however available from the authors upon reasonable request and with permission of the local ethics committee of MHS.

## Abbreviations

GP: General practitioner
IC: Intensive counselling
NRT: Nicotine replacement therapy
NS: Non-significant
HMOs: Health maintenance organizations
MHS: Maccabi Healthcare Services
SES: Socioeconomic status
DMt2: Diabetes mellitus type 2
IHD: Ischemic heart disease
COPD: Chronic obstructive pulmonary disease
OR: Crude odds ratio
aOR: Adjusted odds ratio
